# Defects Prediction Method for Radiographic Images Based on Random PSO Using Regional Fluctuation Sensitivity

**DOI:** 10.3390/s23125679

**Published:** 2023-06-17

**Authors:** Zhongyu Shang, Bing Li, Lei Chen, Lei Zhang

**Affiliations:** 1 State Key Laboratory for Manufacturing Systems Engineering, Xi’an Jiaotong University, Xi’an 710054, China; zshang@stu.xjtu.edu.cn (Z.S.); lb@xjtu.edu.cn (B.L.); zl1872083252@stu.xjtu.edu.cn (L.Z.); 2 International Joint Research Laboratory for Micro/Nano Manufacturing and Measurement Technologies, Xi’an Jiaotong University, Xi’an 710049, China

**Keywords:** PSO, defect prediction, image processing, radiographic testing, turbine blades

## Abstract

This paper presents an advanced methodology for defect prediction in radiographic images, predicated on a refined particle swarm optimization (PSO) algorithm with an emphasis on fluctuation sensitivity. Conventional PSO models with stable velocity are often beleaguered with challenges in precisely pinpointing defect regions in radiographic images, attributable to the lack of a defect-centric approach and the propensity for premature convergence. The proposed fluctuation-sensitive particle swarm optimization (FS-PSO) model, distinguished by an approximate 40% increase in particle entrapment within defect areas and an expedited convergence rate, necessitates a maximal additional time consumption of only 2.28%. The model, also characterized by reduced chaotic swarm movement, enhances efficiency through the modulation of movement intensity concomitant with the escalation in swarm size. The FS-PSO algorithm’s performance was rigorously evaluated via a series of simulations and practical blade experiments. The empirical findings evince that the FS-PSO model substantially outperforms the conventional stable velocity model, particularly in terms of shape retention in defect extraction.

## 1. Introduction

Particle swarm optimization (PSO) is a global optimization method first proposed by Kennedy and Eberhart in 1995 [1]. Inspired by collective behavior in nature, PSO has become a widely used technique in various fields. Initially designed for optimizing continuous nonlinear functions, the original PSO algorithm has undergone numerous enhancements to tackle complex problems [2]. These modifications enable its application in multiobjective, constrained, discrete, and binary optimization scenarios [3]. In imaging processing fields, there are some notable applications using the PSO algorithm:

- Clustering and image segmentation (classic PSO model);
- Multilevel image thresholding (modified PSO model);
- Noise reduction (modified PSO model);
- Evolving deep convolutional neural networks (hybrid PSO model).

Despite its improved performance, the PSO algorithm still faces challenges such as convergence speed, premature convergence, sensitivity to initial values, and manual parameter determination. This section explores the significance of PSO architecture design, considering various modifications in fitness functions and velocity configurations, as demonstrated through industrial applications.

### 1.1. PSO Applications in Image Processing

The wide range of applications and advancements in the PSO algorithm highlight the importance of PSO architecture. Initially, PSO research focused on clustering, classification, and data mining from uncertain image datasets, outperforming traditional methods [4,5]. Image segmentation benefits from PSO’s ability to determine the number of clusters autonomously using a histogram [6,7], while image thresholding reduces tolerances caused by uneven distribution curves [8]. PSO has also been integrated with other methods, such as SVM for surface defect detection and wavelets for medical image compression [9,10]. Hybrid approaches, such as PSO-K-means for MRI segmentation, PSO with machine learning for image enhancement, and PSO with a residual network for pipeline robot fault diagnosis, further improve performance [11,12,13,14]. In conclusion, various PSO models designed for specific applications have their respective advantages and disadvantages. These include:

- Classic and Modified PSO Model

The original PSO model offers significant ease of use. By replacing the fitness function with a specific mathematical equation or method, the classic model can be seamlessly integrated into nearly any application scenario. On the other hand, PSO modifications, such as evolving weights during iterations, offer greater probability for PSO implementations in complex images, such as multilevel thresholding. However, such architecture is limited for complex problems due to the lack of swarm diversities.

- Hybrid PSO Applications

Owing to its iterative features, the PSO algorithm is ideal for resolving the uncertainty inherent in images. This attribute facilitates the acceleration of the optimization process in numerous hybrid applications. However, controlling convergence during iterations is a formidable challenge in the hybrid model. This is because the hybrid model, laden with numerous functions and structures, may trigger premature convergence, thereby preventing the global optimal value from reaching the anticipated result post all iterations.

### 1.2. Defect Detection in Radiographic Images with PSO Implementations

Combined with deep learning and other machine learning methods, PSO offers a robust approach for defect detection and classification in radiographic images. Unlike traditional models, PSO provides flexibility in feature selection and classifier optimization, resulting in improved performance and reduced computational load. Defect detection in radiographic images is crucial for safety and reliability in industries such as inline inspection and medicine [15,16]. Recent research focuses on learning-based models such as deep learning, SVM, and other machine learning methods for feature extraction, selection, and classification. For instance, Dias Júnior et al. [17] achieved high accuracy in classifying COVID-19 patients using PSO-optimized XGBoost with deep features. Narin [18] applied PSO for feature selection in CNN models, achieving exceptional performance. Kumari et al. [19] proposed a hybrid algorithm for segmenting COVID-19 infected X-ray images using PSO and K-means. Açıcı et al. [20] used PSO and GA to optimize CNN hyperparameters for femoral neck fracture detection. In weld defect detection, Ma et al. [21] achieved high accuracy using machine learning and active visual sensing. Naddaf-Sh et al. [22] trained an optimized CNN for detecting weld defects. Hena et al. [23] emphasized the importance of signal-to-noise and contrast-to-noise ratios in deep learning for NDT applications.

### 1.3. PSO Fitness Function and Velocity Configuration for Defects Analysis

CPSO-based methodologies show promise in defect detection and prediction. The design of an optimal fitness function and velocity setting is crucial for efficient PSO-based implementations.

However, challenges exist in PSO-based approaches. The fitness function plays a key role in defect detection, requiring the ability to differentiate between defective and nondefective areas and identify various types of defects [24]. For instance, in a study on leather defect detection, a modified fitness function using selective-band Shannon entropy improved segmentation efficiency [25]. The velocity setting also influences algorithm convergence and search efficiency [26]. Balancing exploration and exploitation behaviors, the velocity should facilitate effective solution space exploration without being trapped in local optima. Some studies propose enhancements to the velocity updating process, such as incorporating Lévy flight strategy [27].

Despite the advantages of PSO in defect analysis, there are certain limitations:

- PSO approaches often entail a high computational cost due to a large number of iterations [28].
- Selecting appropriate parameters for the PSO algorithm is challenging, often requiring specific fitness function designs for different problem domains [29].
- Premature convergence leading to local optima is a risk. Velocity configuration can enhance swarm diversity and behavior during iterations to mitigate this issue [30].

This paper introduces a novel variant of the PSO algorithm, which incorporates regional fluctuation sensitivity for defect prediction in radiographic images. The proposed modification of the PSO algorithm utilizes spatial entropy and an evolving swarm velocity to accurately identify defect regions based on regional fluctuation phenomena. This unique approach aims to circumvent the prevalent issues of premature convergence and elevated computational expense, commonly encountered during the defect tracing process. Through the theoretical model, simulation, and actual experiment, the result consists of three main outcomes:

1. The proposed PSO model has lower computation cost than the stable velocity PSO model.
2. Premature convergence is mitigated and optimized by velocity configuration.
3. Traced defect areas have significantly higher shape retention than the stable velocity PSO model.

Section 2 establishes the relationship between regional entropy features and fluctuation phenomena for defect prediction. Section 3 outlines the structure design of velocity and the fitness function critical for the defect tracing procedure. A simulation experiment with a turbine blade model embedded with artificial defects validates the defect prediction performance in Section 4. Section 5 presents laboratory-based experimental results using an actual turbine blade, examining performance, computational efficiency, convergence analysis, and the correlation between PSO results and defect dimensions. Finally, Section 6 summarizes the findings, evaluates them, and suggests directions for future research.

## 2. Related Work

### 2.1. PSO Fitness Function Design Based on Entropy Theory

Entropy theory has been effectively applied in image processing, including the use of PSO models for edge detection, segmentation, and thresholding [31,32,33]. Despite its ability to optimize global values by analyzing regional variations, PSO fails to detect defects on a global statistical scale. Therefore, the integration of entropy in PSO tracking necessitates both its use as a fitness function and a comprehensive modification of PSO for adaptive defect detection.

Radiography images exhibit a wide dynamic range (usually 14–16 bit) and high resolution in the exposure orientation. The pixel information in these images can provide a comprehensive understanding of potential defects. However, traditional thresholding methods are not suitable due to irregular gray value discrepancies within defect regions. The entropy algorithm offers a solution by utilizing regional statistical methods to calculate entropy values based on the relativity of neighboring pixel values.

The entropy algorithm seeks to identify defects by employing regional statistical methods to calculate the target area based on the relativity of neighboring pixel values. The calculation process for entropy J, as illustrated in Equation (1), utilizes the target pixel (i,j) value $I_{N}$ along with N neighbors in an image with an M-bit depth.

(1) $$J(i,j)=\sum_{0}^{2^{M}-1}I_{N}(i,j)log_{2}I_{N}(i,j)$$

Within the defect area, the gray value of neighboring pixels in close proximity to the defect center exhibits significant changes due to uneven density. Theoretically, the mathematical value of entropy J(i,j) reflects the regional abundance within the target area. This regional feature renders the entropy algorithm suitable for the precise detection of defects in radiographic images. The relationship between exposure settings and image quality relies on a quantified method to determine whether the actual data are optimal for detection purposes. In contrast to the International Electrotechnical Commission (IEC) standard guidelines [34,35], conventional exposure indicators employ a preset reference for exposure settings as directed by the manufacturer. Conversely, the entropy algorithm offers a quantified approach to assess image quality, utilizing abundance as a reference for specific tested objects.

**Figure 1:**
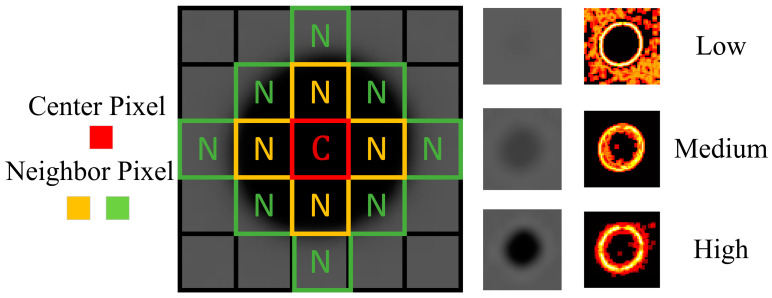
Entropy computation at the defect area with different values in exposure images.

Figure 1 illustrates the entropy features at the defect area, demonstrating how the entropy value increases with the contrast under different exposure settings. Excessive exposure energy penetrating through the defect can cause indistinguishable gray values from neighboring pixels, resulting in a decrease in calculated entropy. Therefore, the highest entropy value guides the determination of the optimal exposure setting for defect detection. This entropy-based method offers a valuable alternative to conventional exposure indicators that rely on preset references. Instead, it leverages the abundance of information within the target pixel area to assess image quality in radiographic defect detection.

### 2.2. Fluctuation Phenomenon of Entropy Value in Defect Area

During radiographic imaging, scattering effects caused by small gaps and edges in tested objects result in blurred edges in the image [36,37]. This phenomenon arises from multiple reflections in small corners, amplifying regional X-ray intensity [38]. The area surrounding these gaps exhibits inconsistent gray values and reduced accuracy, making it conducive to defect detection. Defects such as cracks or holes introduce regional enhanced intensity effects, leading to fluctuations in gray values [39,40]. Entropy theory suggests that increased regional pixel value abundance corresponds to higher entropy values, allowing identification of regional fluctuations caused by defects.

**Figure 2:**
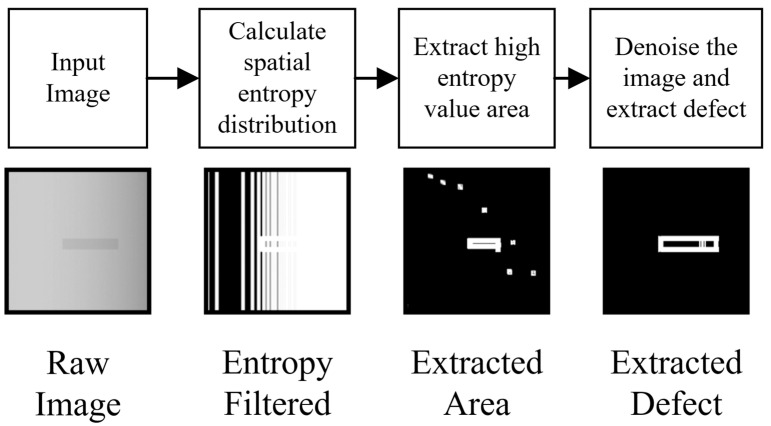
Entropy extraction procedure by regional fluctuation at two different defect locations.

Figure 2 demonstrates the procedure for extracting defects using the entropy filter. The defect hole area exhibits a significantly higher entropy value than other areas through the filter calculation. After thresholding the extracted area based on the defect’s entropy value and denoising, the defect area can be precisely detected.

### 2.3. PSO with Fluctuation Sensitive Invariant

In practical scenarios, defects often exhibit a random distribution within tested objects. The PSO algorithm, with its random optimization process, is suitable for identifying regions with fluctuations. Traditional setups tend to attract particles towards the highest gray value when using a gray value thresholding method in the fitness equation. However, if the defect area’s gray value is obscured by complex geometric objects, such as free-form surfaces, detecting the defect becomes challenging. Implementing entropy, as explained in Equation (1) and demonstrated in Figure 1, enables more effective highlighting of regional fluctuations through statistical calculations, making it suitable for the fitness function.

PSO employs random particles with predefined velocities to search for the target fitness value at specific locations. In each iteration, guided by the fitness equation, the global optimal position gradually approaches the defect area due to the magnification effect of regional fluctuations on the particles. This effect arises from uneven density within the defect area, where the biased thickness is amplified through each PSO iteration.

**Figure 3:**
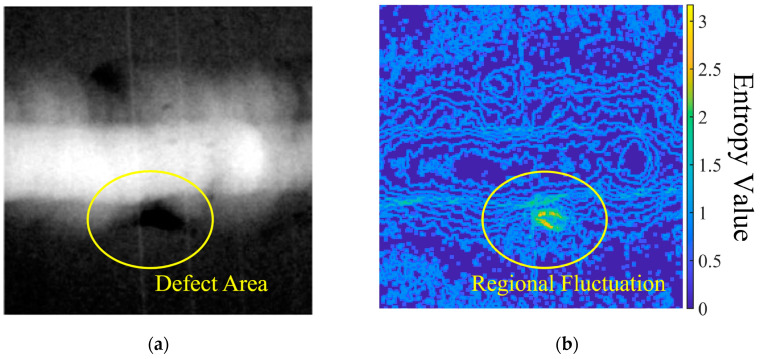
Entropy filter comparison with (**a**) raw image (contrast adjusted for visibility); (**b**) filtered image with highlighted regional fluctuation.

Figure 3a displays a typical radiographic image of a welding point in a gas pipeline with a void defect. The image is processed with Equation (1) taking N=9 to obtain the entropy filtered image in Figure 3b. According to the indicated color map, the entropy distribution in the defect area is significantly higher (J≥3) compared to neighboring pixels (J≤1.5).

By utilizing entropy value as the fitness equation, PSO demonstrates fluctuation sensitivity characteristics. This method is ideal for potential defect recognition, offering adaptive detection capabilities for tested objects. The optimized locations identified through PSO iterations demarcate the target area of the defect within a specific region.

## 3. PSO Algorithm Structure Design

### 3.1. Flexible Velocity Design Based on Thresholding

Conventional defect detection methods use thresholding processes to identify potential defect areas, but they often face limitations in handling regional defects with irregular shapes. To overcome threshold limitations, an optimization strategy for constraining particle velocity is introduced.

(2) $$V_{particle}=V_{PSO}\times w_{s}\times (I_{ref}{-I}_{particle})$$

(3) $$w_{s}=w_{g}\times ln\frac{I_{ref}}{V_{max}}$$

(4) $$\underset{I_{\mathrm{particle}}\to I_{\mathrm{ref}}}{\lim}V_{particle}=0\;\underset{I_{\mathrm{particle}}\to 0}{\lim}V_{particle}=V_{PSO}\times w_{s}\times I_{ref}$$

Equation (2) outlines the basic procedure for velocity setting. $V_{PSO}$ refers to the stable velocity model for velocity setup [41], while $I_{ref}$ represents the reference grayscale threshold. The coefficient $w_{s}$ is employed to set the velocity based on the difference between the threshold value $I_{ref}$ and the particle value $I_{particle}$. In Equation (3), the coefficient $w_{s}$ consists of the division of $I_{ref}$ and $V_{max}$, along with a constant $w_{g}$. Equation (4) describes two limit values of particle velocity $V_{particle}$, which is the velocity range set by the coefficient $w_{s}$ and the gray value of the particle location ($I_{particle})$. Notably, $V_{particle}$ might be set as a negative value due to bidirectional movements in the image’s space.

During the PSO process, particles generated at random pixel locations are assigned velocities according to the gray value difference between the threshold and the pixel itself. As the difference decreases, the velocity increases. The relationship between velocity $V_{particle}$ and difference is adjusted by $w_{s}$, which restricts the intensity of particle movements. The purpose of coefficient $w_{s}$ is to provide the particle with sensitivity for detecting regional fluctuation variations. When a particle is situated at the edge of a defect area, the difference between $I_{ref}$ and $I_{particle}$ will significantly increase. Consequently, the velocity $V_{particle}$ decreases, slowing the particle’s movement and trapping it within the defect area. Moreover, particles have a random chance to move to the edge of any area in the image. In this situation, regular-shaped areas without defects will assign $V_{particle}$ a significantly high value, causing the particle to flee from the current area during the next iteration.

### 3.2. PSO Structure Integrated with Entropy Fitness Function

The proposed PSO model employs the pixel’s entropy value as the fitness function for evaluating regional fluctuations to predict potential defect areas.

**Figure 4:**
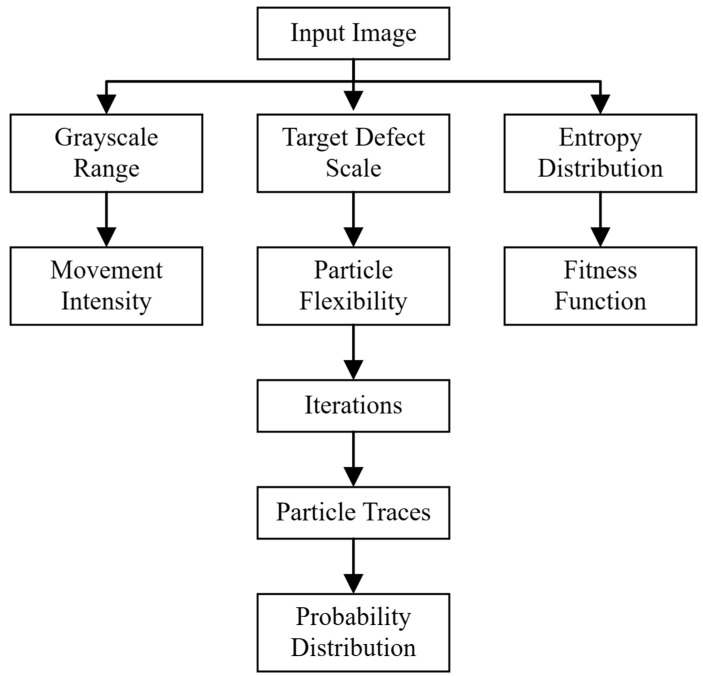
Workflow of defect prediction using PSO algorithm.

Figure 4 depicts the workflow of the FS-PSO (fluctuation-sensitive) algorithm. Based on the input image and target defect scale, the intensity coefficient $w_{s}$, including reference threshold $I_{ref}$ and velocity limit $V_{max}$, is established to indicate expected defect predictions with a specific movement intensity. The fitness function pertains to the spatial entropy distribution of the input image, where the regional fluctuation is enhanced, as demonstrated in Figure 3. Upon implementing the PSO process, a probability distribution map is generated from the optimal particle statistics.

### 3.3. Defect Prediction Method for Radiographic Testing

The PSO process applied to radiographic images extracts a set of locations converging towards the theoretical center of the fluctuation area. To quantify potential defects, these optimal locations necessitate a statistical model to address the probability distribution in the image.

(5) $$P[i,j]=\frac{\sum_{0}^{n}N_{particles}}{\left[i_{target}\times j_{target}\right]}$$

(6) $$\underset{N_{\mathrm{particles}}\to n}{\lim}P[i,j]=100\%$$

The probability of a defect in the pixel matrix $\left[i_{target}\times j_{target}\right]$ is depicted in Equation (5), calculated by dividing the number of optimal particles by the total pixel number of the matrix $\left[i_{target}\times j_{target}\right]$ in the region. Equation (6) demonstrates the situation when all particles are located within the pixel matrix, which indicates the defect area. Employing the probability method for defect prediction description helps circumvent the background interference issue, which arises when objects with free-form geometry exhibit high entropy values in their spatial distribution.

As the size of potential defects is relatively small compared to the tested object, the probability prediction serves as an approachable method, utilizing the PSO iteration algorithm to filter interferences instead of relying on conventional thresholding methods.

## 4. Simulation Results

### 4.1. PSO Tracing Implementation on Theoretical Model

**Figure 5:**
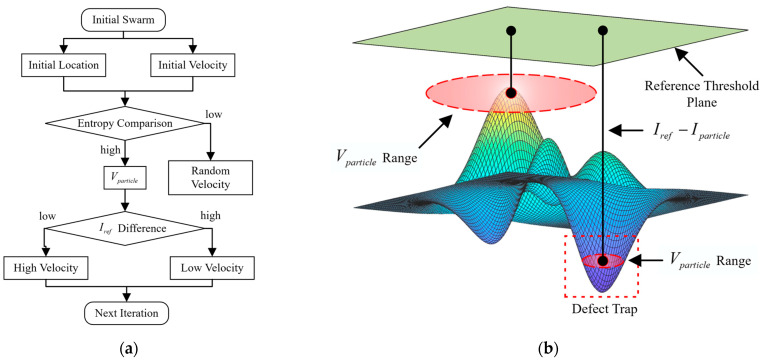
FS-PSO tracing framework in (**a**) workflow in a single iteration; (**b**) defect trap mechanism.

The proposed PSO model, featuring fluctuation invariance, is illustrated in Figure 5a. Upon generating the particle swarm, each particle’s velocity is constrained by the global entropy value of the input image. If the entropy value at the particle’s location is lower than the global value, the velocity is set randomly for further movements. Conversely, if the pixel entropy at the particle’s location exceeds the global value, the velocity is determined based on the value difference with the reference threshold $I_{ref}$. Figure 5b showcases the defect-trapping mechanism facilitated by the PSO algorithm with fluctuation invariance. When a particle enters a defect with a lower gray value, its velocity is significantly reduced, causing the particle to be trapped within the defect area. After several iterations, numerous particles become clustered within the defect area.

**Figure 6:**
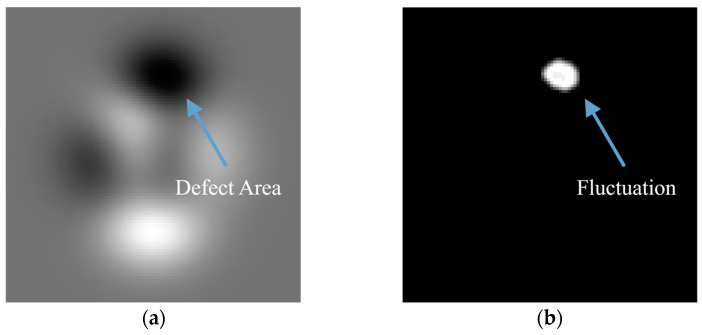
Regional fluctuation analysis on parabolic polynomial equation in (**a**) simulated image; (**b**) spatial entropy.

In accordance with the presented method, a simulated curved surface using a parabolic polynomial equation is employed for testing. The designated defect area exhibits relatively lower gray values compared to the surrounding areas. Figure 6 portrays this phenomenon. When entropy calculation is applied, the defect area exhibits a peak entropy value, indicative of regional fluctuation.

### 4.2. PSO Result on Radiographic Image

**Figure 7:**
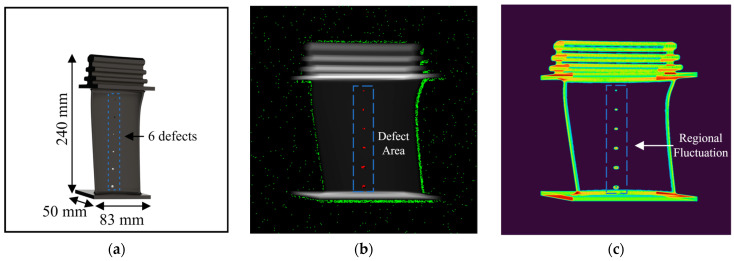
Blade model and simulation results in (**a**) blade model; (**b**) particle racing result in radiographic image; (**c**) spatial fitness function (spatial entropy).

The PSO experiment employs a radiographic image of a free-form turbine blade model generated using the voxel method [42,43,44]. Figure 7a,b display the blade model, which has dimensions of 50 × 83 × 240 mm and features six artificial defect holes, detailed in Table 1. The exposure distance is set to 1 m, and the grayscale image has a resolution of 1557 × 1557 with a 14-bit depth. The swarm consists of 5000 particles, and the experiment runs for 100 iterations. Multiple attempts are conducted to ensure the accuracy of the PSO results.

Figure 7b,c illustrate the PSO tracing results, with particle traps marked by red dots in (a). As the fitness function utilizing fluctuations exhibits higher values around the defect area, particles traversing these regions become ensnared. Additionally, due to the decreasing velocity at lower gray values, particles situated in the background with minimal fluctuations and entropy values also become trapped after a certain number of iterations.

### 4.3. Iteration Analysis on Velocity Invariant

**Figure 8:**
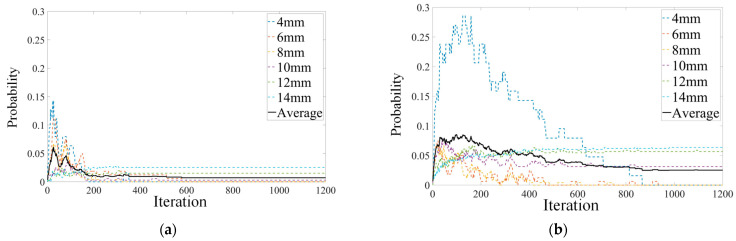
Comparison of random invariant in (**a**) no random invariant; (**b**) 10% random invariant integrated in high $I_{ref}$ difference.

In PSO algorithm structure design (Section 3.1), the PSO structure is designed with fluctuation sensitivity for defect trap functions. However, movement intensity also influences the particle swarm. For example, if the intensity is too low, the particle velocity would be low, making it difficult for particles outside the defect trap to seek another trap location. In such cases, the number of particles falling into the defect area will decrease. Figure 8 demonstrates the PSO process with 10% random invariant integration on particle velocity.

In Figure 8a, the velocity set in each iteration without an implemented random variant exhibits faster convergence speed. The swarm reaches a relatively stable state at around 600 iterations, with the probability of particles inside the defect trap at approximately 0.01 (1% of the total swarm). In Figure 8b, the velocity is assigned a random value if the difference between $I_{particle}$ and $I_{ref}$ is high. Under this condition, particles exhibit greater movement intensity rather than a linear relationship with the reference threshold value during each iteration. The results reveal that the overall probability of particles within the defect area stabilizes at around 0.03 (3% of the total swarm) within 900 iterations. Consequently, the random variant application can enhance the defect trap function but may reduce the efficiency of the PSO tracing process.

### 4.4. Defect Prediction with Indicated Dimension

**Figure 9:**
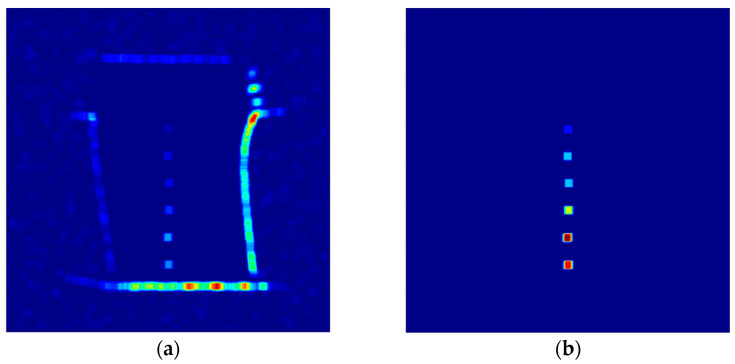
Defect prediction distribution in (**a**) probability map; (**b**) filtered map.

To verify the PSO tracing results and generate the probability map for defect prediction, the presented method employs a detection window of 20 × 20 pixels to calculate the probability P[i,j] in Equation (5). The PSO tracing process utilizes 1000 particles and 100 iterations for training, with the experimental results obtained by averaging values from 1000 repeated processes. Figure 9 presents the processed probability map, and the PSO statistical results are listed in Table 2.

Based on the results, the average number of particles trapped within the defect area is approximately 70.36, covering 3.69% of the total pixels in the defect area. According to the varying sizes of the six defects, the trapped particles exhibit an incremental trend in each defect area, as the regional entropy value is directly proportional to the gray value fluctuations.

## 5. Experimental Results

### 5.1. Experimental Result on Turbine Blade Radiographic Image

**Figure 10:**
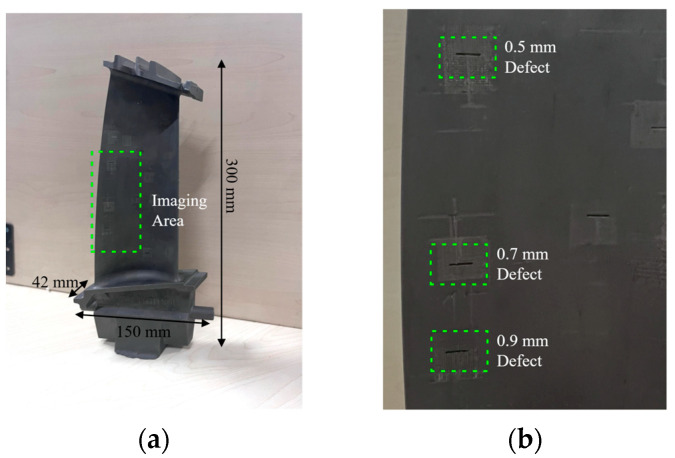
Test turbine blade for experiment in (**a**) overall dimension; (**b**) details of three artificial defects.

**Figure 11:**
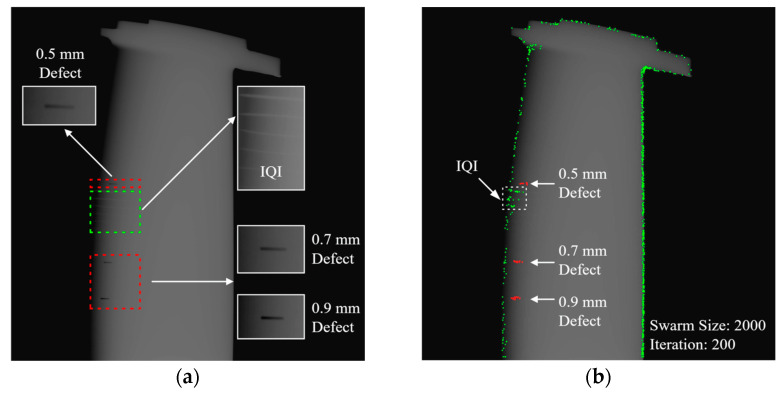
Radiographic image of test blade in (**a**) raw image; (**b**) FS-PSO process result.

Following the validation of the simulation blade model using the PSO tracing method, an experiment involving an actual blade is conducted to further validate the defect prediction model. Figure 10 displays the utilized nickel-based alloy blade, which features a set of artificial defects. The specifications of the in-lab radiography system include a 225 kV X-ray source and a 14-bit flat panel detector with a 222 mm × 222 mm imaging window, 1557 × 1557 resolution, and 143 μm pixel pitch. The experiment employs three defects with dimensions of 5 mm length and 0.5 mm width for radiographic imaging. Each defect has a depth of 0.5 mm, 0.7 mm, and 0.9 mm, respectively. The exposure setup utilizes an ASTM 1A6 image quality indicator (IQI) affixed to the blade to ensure optimal image contrast in the imaging area. The resulting tested image is presented in Figure 11.

The PSO tracing experiment setup involves 200 iterations and various particle sizes, ranging from 2000 to 10,000, with the average result generated from 1000 repetitions. Figure 11b displays the PSO results with a 2000-swarm setting, where particles in the defect area are marked in red. Table 3 lists the PSO results under different settings generated by Intel Xeon workstation equipped with an E5 2670 V2 dual CPU, 128 GB DDR3 RAM, Nvidia RTX 3080 Ti graphic card. The swarm is filtered by eliminating particles in the background with a zero-entropy value. As the swarm size increases, the ratio of total particles trapped in the defect area also rises.

### 5.2. Relation of Probability Prediction with Defect Dimension

**Figure 12:**
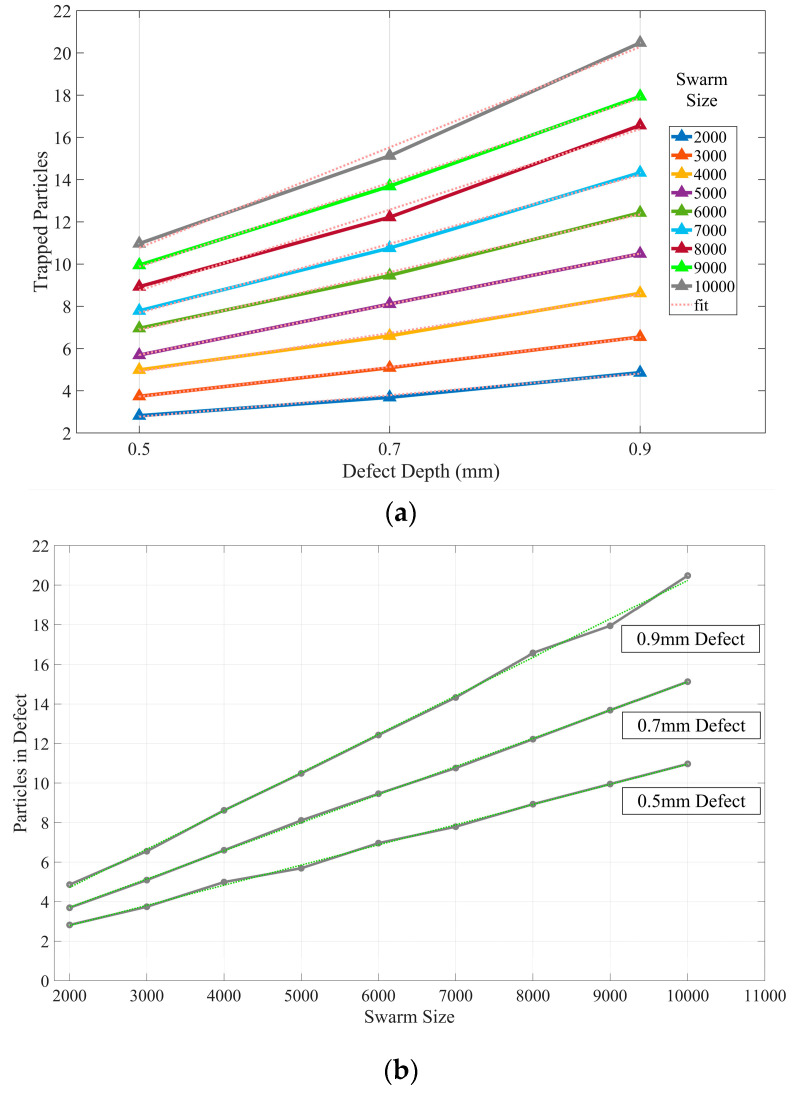
Experimental results analysis in (**a**) relation of defect depth and trapped particles; (**b**) relation of swarm size and particles in defect.

Figure 12 depicts the particles trapped in each defect with different swarm sizes, utilizing the same settings as in Section 5.1. As deeper defects exhibit higher regional entropy values, the PSO tracing method demonstrates optimal performance in the 0.9 mm defect area. In Figure 12, the increment in swarm size exhibits an approximately linear relationship. With the same input image in the PSO algorithm, the slope of the curve correlates with the defect’s depth since deeper defects exert greater influence on particle velocity, as per Equation (2). Nonetheless, setting an excessively large swarm size would increase overall computational demands.

### 5.3. Convergence Behavior on Velocity Intensity

**Figure 13:**
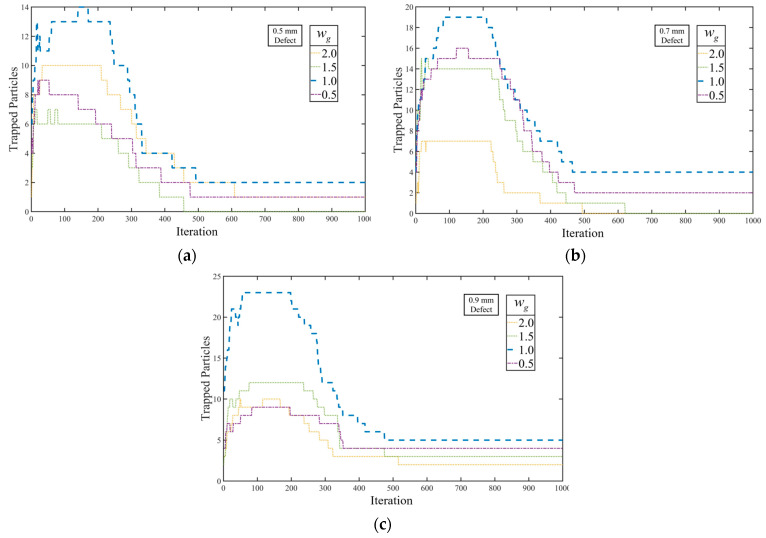
Influences on FS-PSO convergence in (**a**) 0.5 mm defect; (**b**) 0.7 mm defect; (**c**) 0.9 mm defect.

The performance of the proposed PSO algorithm is evaluated under different velocity settings to examine the impact of $w_{g}$ on the tracing procedure. The experiment is designed with a swarm size of 10,000 and a velocity limit of 16 pixels (approximately 1/100 image resolution). Figure 13 presents the experimental results for various $w_{g}$ values.

In the tracing results, particles trapped in all defects are sensitive to $w_{g}$ values, which alter velocity intensity and induce chaotic movements in the swarm. As illustrated in Figure 13a–c, any changes in $w_{g}$ decrease the hit rate during initial tracing phases, due to the modification of the linear relationship between regional entropy and velocity limit. Furthermore, when $w_{g}$ is set higher than 1.0, the trapping effects on particles in thinner defects tend to malfunction, resulting in trapped particles escaping the defect area.

### 5.4. Performance Comparison on Defects Predication

To assess the performance of trap effect in the proposed PSO with fluctuation sensitivity (FS-PSO), a comparative analysis was conducted with the conventional PSO technique. The experimental setup comprised 200 iterations and was repeated 1000 times. In the PSO model with stable velocity, the inertia weight $w_{s}$ was assigned a value of 0.9, while the coefficients $\varphi_{1}$ and $\varphi_{2}$ were set to 1.5, in accordance with the algorithmic structure suggested by Kennedy [45]. Both the proposed and stable velocity methods utilized regional entropy as the fitness function for particle tracing tests. The outcomes of the experiment are summarized in Table 4. The computational time of the FS-PSO employing a 10% randomization exhibits a marginally elevated duration, ranging from 1.20% to 2.28% in comparison to the stable velocity model. Notably, this discrepancy diminishes as the swarm size escalates.

**Figure 14:**
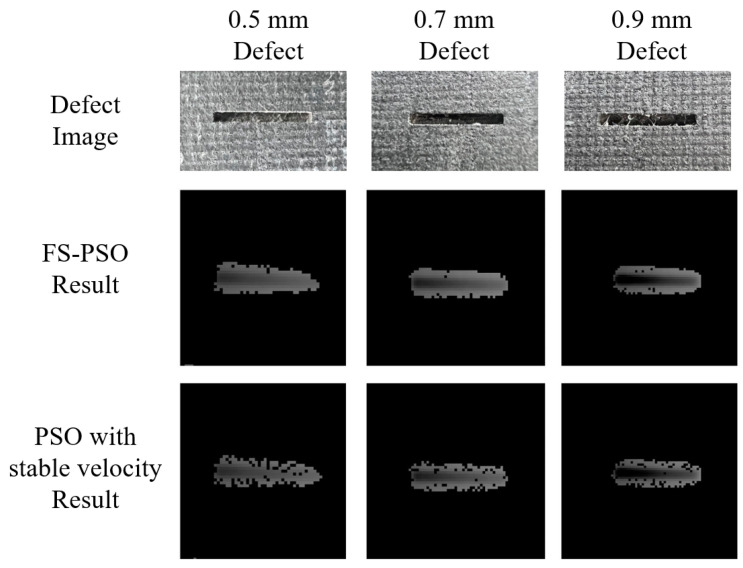
Comparison of defects extraction (100,000 swarm size, 200 iterations) with different PSO models.

Figure 14 presents a comparative analysis of defect extraction employing FS-PSO and PSO with a stable velocity configuration. Owing to the trapping effect inherent in the FS-PSO approach, all defect regions are accurately identified and recovered with high shape retention. In contrast, the PSO model with a stable velocity setting exhibits increased randomness in particle movements during the tracing process, resulting in a more dispersed extraction of defect areas, particularly in cases of larger iteration counts.

Given the outlined limitations of the PSO implementation, as discussed in Section 1.3, it is noteworthy to mention that the proposed FS-PSO algorithm exerts minimal impact on the computational cost. This is primarily due to the velocity configuration being associated solely with the regional entropy value, eliminating the need for high-order computations characteristic of other learning-based models. Furthermore, the regional entropy feature is a statistical value that is universally applicable to all images. Notably, the velocity component of the FS-PSO algorithm, which incorporates a trapped effect, alleviates the local optima problem typically encountered with the stable velocity PSO model. Because the presence of a fluctuation phenomenon is uniquely observed within defect areas, this enhances the model’s ability to accurately identify defects; consequently, the FS-PSO algorithm demonstrates superior efficacy in the optimization of image processing tasks, underlining its practical utility in this domain.

## 6. Discussion

This section presents a parallel comparison and analysis of the proposed FS-PSO model and the stable velocity model, primarily to evaluate the overall performance and architectural advances of the FS-PSO model. For a parallel comparison, both models employ the same randomly generated swarm of 2000 particles. As indicated by the data in Figure 13, the number of iterations is set at 200 to prevent premature convergence.

**Figure 15:**
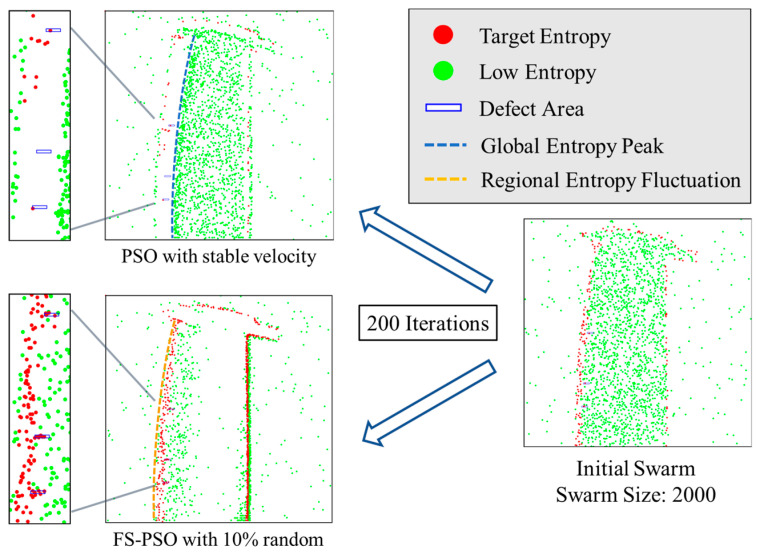
Swarm behavior analysis of FS-PSO and stable velocity model using same initial swarm.

Figure 15 provides a demonstration of swarm behavior within both models. Particles represented by red dots indicate a target entropy value higher than global entropy, while those represented by green dots denote a lower entropy value. Notably, the convergence speed of the FS-PSO model is considerably faster, while the swarm in stable velocity model is stuck without further movement. In the FS-PSO model, the swarm bifurcates into two smaller swarms. One swarm gravitates toward the blade’s edge areas due to high entropy value, while the other moves within the blade’s thinner region, progressively drawn toward defects. In the stable velocity model, the swarm is attracted to the peak spatial entropy value due to the entropy fitness function. After 200 iterations, the majority of the particles have migrated toward areas of high entropy, demarcated by a blue dotted curve in Figure 15.

Such differences in the two models are caused by the free-form surface of the tested blade, which have been discussed in previous studies [46,47,48]. Because of the significant thickness variation in free-form objects, radiographic images are obtained using compromised exposure settings to capture all possible data. Consequently, the thicker area in the blade image appears underexposed due to limited X-ray energy, resulting in a high-entropy boundary (blue dotted curve in Figure 15), acting as an edge effect. Conversely, the thinner region, which receives abundant X-ray energy, contains more pixel information, thereby creating a larger regional entropy value (orange dotted curve in Figure 15) conducive to the trap effect utilized by FS-PSO for defect tracing.

As a result, as evidenced in Table 4 within Section 5.4, the FS-PSO model demonstrates a substantial augmentation of around 40% in particle entrapment within defect areas when contrasted with the stable velocity model. Furthermore, the swarm within the FS-PSO model exhibits notably reduced chaotic movement patterns. This indicates that, despite the variation in defect tracing between both models ranging from 10% to 60%, as per Table 4, the actual increase in defect-bound particles results from diminished movement intensity as the swarm size increases.

While the FS-PSO model focuses on regional entropy fluctuations, it demonstrates superior convergence speed in regional feature extraction compared to the stable velocity method. Despite the stable velocity model’s proficiency in rapid segmentation and thresholding, with an emphasis on global image features validated by our prior work, the comparison experiment supports that the FS-PSO model exhibits remarkable shape retention in defect recovery, a feature attributable to its regional trap capabilities. Key features of the FS-PSO model are summarized as follows:

- Low computation cost: Though the mathematical complexity of FS-PSO is slightly greater than that of stable velocity models, it exhibits a linear computation cost relative to its swarm size, which is marginally higher than stable velocity models.
- Adaptive architecture for defect prediction: The velocity configuration of trap effects in FS-PSO alters swarm behavior to prevent premature convergence during iteration procedures.
- Local optima mitigation: With a direct focus on regional fluctuations for defect tracing, the FS-PSO model exhibits a faster convergence process compared to the stable velocity model, mitigating any local impasses.

## 7. Conclusions and Future Work

This paper proposed an improved defect prediction method for radiographic images based on PSO architecture design referring to fluctuation sensitivity. Characterized by approximately 40% enhanced particle entrapment within defect areas, faster convergence speed, and less chaotic swarm movement, the FS-PSO model improved efficiency by altering movement intensity as swarm size increased. Despite a slight increase in mathematical complexity, the model maintains a linear computation cost. Its adaptive architecture, featuring a velocity configuration that adjusts swarm behavior, effectively curtails premature convergence while also successfully mitigating local optima by guiding particle movements toward regional fluctuations for defect tracing.

Despite the successful application of regional fluctuation for velocity adjustments to address the issue of premature convergence in the context of locating small defects, the characterization of these defects remains an unresolved problem. While Section 5.2 demonstrated a linear relationship between the predicted probability and defect dimensions, developing a precise model for three-dimensional defect characterization remains a substantial task. Although our prior research has delved into defect characterization, the implementation of this process using a single two-dimensional image presents multiple challenges. These include the spectrum analysis of X-ray imaging, calibration of exposure parallax, and optimization of exposure parameters. Consequently, these complexities necessitate further investigation and experimentation within the radiography system.

Future work is mainly separated into two topics. The first topic focuses on continual enhancements to the FS-PSO architecture, particularly towards establishing a correlation between optimal iterations and swarm size. The purpose is to enable the PSO model to become self-adaptive, effectively tracing any potential defects within target objects. The second topic involves optimizing the methodologies of radiographic testing, such as through multiple-exposure testing. This would yield test images that are more compatible with the proposed FS-PSO algorithm, thereby enhancing its efficiency and accuracy.

## Figures and Tables

**Table 1:** Dimension of 6 artificial defects in simulated blade model.

Defect Number (Top to Bottom)	Defect Diameter (mm)	Defect Hole Type	Defect Cone Angle (Degree)	Defect Depth (mm)
1	4	Conical	80.54	5.62
2	6	Through	80.54	5.83
3	8	Through	80.54	5.90
4	10	Through	80.54	5.92
5	12	Through	80.54	5.90
6	14	Through	80.54	5.84

**Table 2:** PSO results on artificial defects in simulated blade model.

Defect Number	Regional Pixels	Average Entropy	Particles in Defect	Probability %
1	63	2.73	3	5.62
2	161	3.58	9	5.83
3	240	3.82	14	5.90
4	352	3.86	21	5.92
5	459	3.78	27	5.90
6	630	3.37	37	5.84
	Total	Average	Total	Average
	1905	3.52	111	3.69

**Table 3:** PSO tracing and computation result of 3 defects with different swarm size.

Total Swarm	Filtered Swarm *	0.5 mm Defect *	0.7 mm Defect *	0.9 mm Defect *	Computation Time (ms) *
2000	626.5	2.82	3.69	4.86	402.5
3000	930.8	3.74	5.09	6.55	430.9
4000	1238.7	4.99	6.60	8.62	455.2
5000	1535.8	5.69	8.11	10.49	475.9
6000	1831.0	6.96	9.46	12.43	494.5
7000	2162.9	7.79	10.76	14.32	511.5
8000	2437.2	8.93	12.22	16.57	562.9
9000	2763.3	9.95	13.69	17.94	582.6
10,000	3042.7	10.97	15.13	20.48	598.8

* The results are generated by the average result from 1000 repetitions using MATLAB R2022b Update 3 9.13.0.2126072 64-bit win64 version.

**Table 4:** Performance comparison of FS-PSO and PSO model with stable velocity settings.

Algorithm Model	Swarm Size	Filtered Swarm *	Trapped Particles *	Computation Time (ms) *
FS-PSO model	2000	606.2	3.17	404.4
	5000	1529.9	17.86	482.3
	8000	2431.2	25.34	560.1
	10,000	2433.5	32.35	613.7
FS-PSO model with 10% random	2000	629.1	4.56	410.9
	5000	1533.3	19.85	486.4
	8000	2433.5	26.14	563.2
	10,000	3049.1	33.40	616.5
PSO model with stable velocity	2000	564.4	2.76	401.7
	5000	1481.3	14.71	479.4
	8000	2398.0	22.99	556.3
	10,000	2985.2	31.22	609.1

* The results are generated by the average result from 1000 repetitions using MATLAB R2022b Update 3 9.13.0.2126072 64-bit win64 version.

## Data Availability

The data that support the findings of this study are available from the corresponding author upon reasonable request.
